# Variants in *MME* are associated with autosomal‐recessive distal hereditary motor neuropathy

**DOI:** 10.1002/acn3.50868

**Published:** 2019-08-20

**Authors:** Daojun Hong, Pu Fang, Sheng Yao, Juanjuan Chen, Xiaolei Zhang, Shuyun Chen, Jingfen Zhang, Dandan Tan, Li Wang, Xinsheng Han, Ling Xin, Yan Wang, Meige Liu, Lu Cong, Shanshan Zhong, Hui Ouyang, Xuguang Gao, Jun Zhang

**Affiliations:** ^1^ Department of Neurology The First Affiliated Hospital of Nanchang University Nanchang China; ^2^ Department of Neurology Peking University People's Hospital Beijing China; ^3^ Department of Neurology The Sixth Medical Center of PLA General Hospital Beijing China; ^4^ Department of Neurology Peking University Shenzhen Hospital Shenzhen China; ^5^ Department of Neurology Shanxi Province People's Hospital Taiyuan China; ^6^ Department of Neurology Affiliated Hospital of Guiyang Medical University Guiyang China; ^7^ Department of Neurology Inner Mongolia Baotou City Central Hospital Baotou China; ^8^ Department of Neurology Affiliated Hospital of Jiujiang Medical College Jiujiang China; ^9^ Department of Neurology Traditional Chinese Medicine Hospital of Lianyungang Lianyungang China; ^10^ Department of Neurology Kaifeng City People's Hospital Kaifeng China; ^11^ Department of Health, Exercise Science, and Recreation Management University of Mississippi University Park Mississippi

## Abstract

**Objective:**

To identify a new genetic cause in patients segregating distal hereditary motor neuropathy (dHMN) with an autosomal recessive pattern.

**Methods:**

Whole‐exome sequencing was conducted in two siblings and was combined with segregation analysis. Additionally, 83 unrelated dHMN patients with unknown genetic cause were screened. RNA analysis was performed using blood lymphocytes and HEK293 cells transfected with mutant plasmids. Immunohistochemistry and Western blot analysis was applied to the nerve tissue. The enzymatic activities of mutant proteins were measured in the cultured cells to verify the pathogenicity of variants.

**Results:**

The clinical features of the patients showed late‐onset phenotype of distal motor neuropathy without sensory involvement. We identified that compound heterozygous variants of c.1342C>T and c.2071_2072delGCinsTT in the membrane metalloendopeptidase (MME) gene co‐segregated with the phenotype in a dHMN family. In an additional group of 83 patients with dHMN, compound heterozygous variants of c.1416+2T>C and c.2027C>T in *MME* were identified in one patient. The splice site variant c.1416+2T>C results in skipping of exon 13. The stop variant c.1342C>T induces mRNA degradation via nonsense‐mediated mRNA decay. Transcript levels of MME in the lymphocytes showed no significant differences between the patients and controls. We also identified that *MME* variants were associated with mild decrease in protein expression in the sural nerve and significant impairments of enzymatic activity.

**Interpretation:**

Variants in the *MME* gene were associated with not only a Charcot‐Marie‐Tooth neuropathy phenotype but also with an autosomal‐recessive dHMN phenotype. Loss of function may play a role in the pathogenesis of dHMN.

## Introduction

Distal hereditary motor neuropathy (dHMN), also known as distal spinal muscular atrophy (SMA), is a group of pure motor neuropathies characterized by progressive distal muscle weakness and atrophy without clinical or electrophysiological sensory abnormalities.^1^ At least 30 genes or loci have been associated with dHMNs with autosomal dominant, autosomal recessive, or X‐linked inheritance.^2^ Autosomal recessive dHMNs have been associated with mutations in the *IGHMBP2*, *PLEKHG5*, *DANJB2*, *SIGMAR1*, *SYT2*, *HSPB1*, *ATM*, *TBCE*, and *VRK1* genes.^3^ However, the underlying genetic variants have yet been discovered in more than one‐half of patients with dHMN.^4^

Membrane metalloendopeptidase (MME), also referred to as neprilysin or neutral endopeptidase (NEP), is a neutral transmembrane endopeptidase that hydrolyses peptides at the amino side of hydrophobic residues and inactivates several peptide hormones.^5^ MME is expressed in a variety of normal tissues, especially is enriched in the peripheral nerves.^6^ The heterozygous mutations in *MME* have been associated with late‐onset axonal Charcot‐Marie‐Tooth neuropathy (CMT2), 7 unspecified polyneuropathy, amyotrophic lateral sclerosis, sensory ataxia, cluster headache,^8^ and dominant spinocerebellar ataxia with neuropathy (SCA43),^9^ whereas homozygous or compound heterozygous mutations in *MME* cause autosomal recessive CMT2.^10^ Several forms of dHMNs exhibit phenotypic overlaps with CMT2 that is a motor‐predominant inherited neuropathy with subclinical sensory involvement.^11^, ^12^, ^13^, ^14^ However, no patients with dHMN phenotype caused by *MME* variants have been reported to date. In this study, we identified that compound heterozygous variants in the *MME* gene were associated with dHMN in two siblings, and further investigated the *MME* variants in 83 unrelated patients with dHMN of unknown genetic causes.

## Subjects and Methods

### Subjects

**Figure 1:**
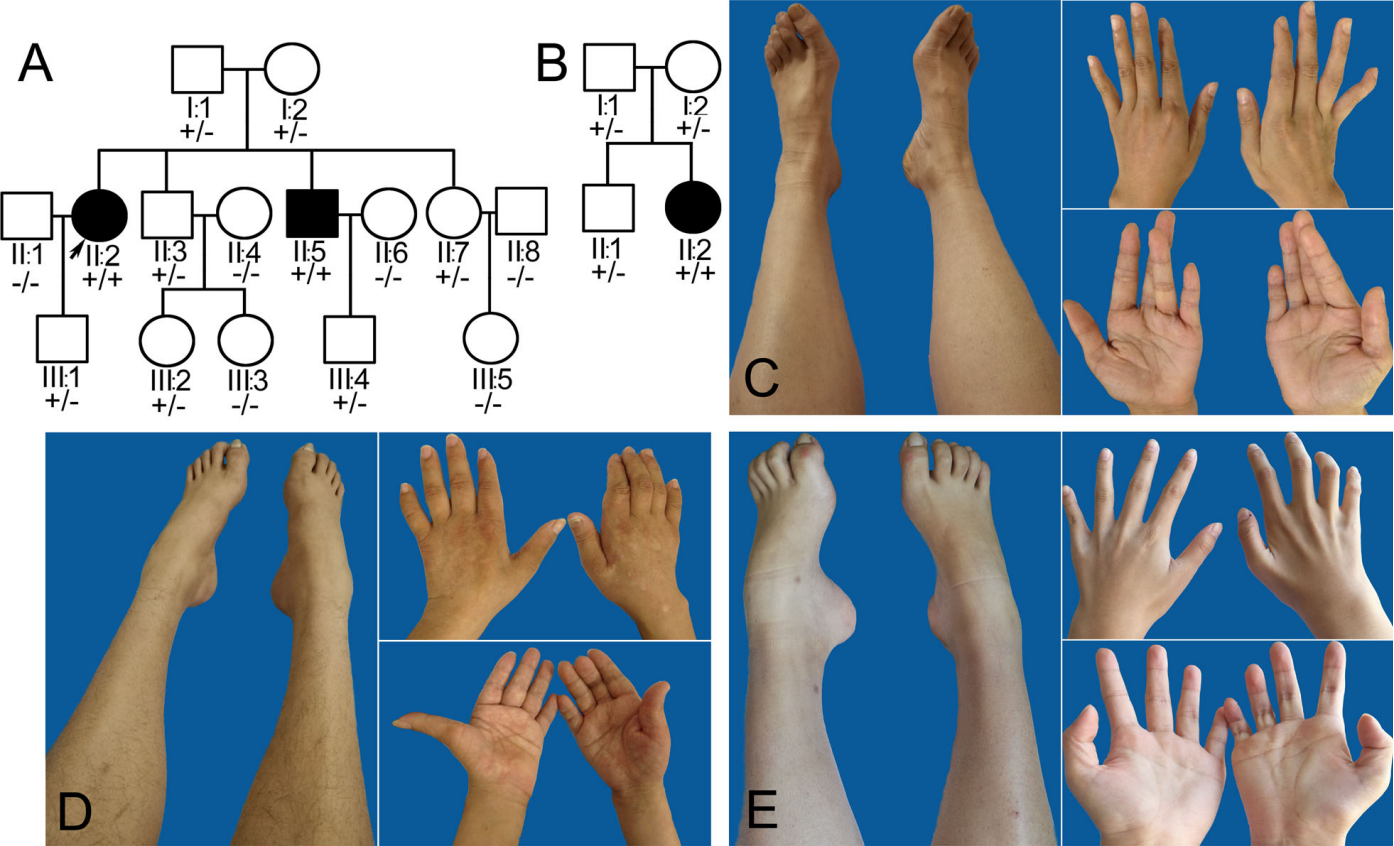
Family pedigrees and illustrations of distal limbs. (A) Family pedigree A shows two patients (arrow indicates the index patient II:2; black square indicates the other patient II:5). The genotype segregates with the phenotype in the family (+ indicates mutant allele; − indicates wild‐type allele). (B) Family pedigree B shows an additional patient II:2 (black circle). Genetic screening reveals a family co‐segregation (+ indicates mutant allele; − indicates wild‐type allele). (C) The patient II:2 of family A shows mild intrinsic muscle atrophy of feet and hands. (D) The patient II:5 of family A has wasting of the lower limbs and left hand. (E) The patient II:2 of family B displays wasting of distal lower limbs and pes cavus.

A nonconsanguineous family A with two affected siblings was initially recruited in this study (Fig. 1A). Clinical evaluations were conducted in 15 members (I:1, I:2, II:1, II:2, II:3, II:4, II:5, II:6, II:7, II:8, III:1, III:2, III:3, III:4, and III:5) from this family by two experienced neurologists. The age at onset, progression of disability, and clinical manifestations were collected. The standard electrophysiological examinations were performed on the affected patients. Brain and spine MRI were also taken on the affected patients. All individuals involved in the study signed written informed consent before they were enrolled in the study. This study was approved by the ethics committees of the Peking University People's Hospital and the First Affiliated Hospital of Nanchang University.

### Exome sequencing

Targeted exon enrichment was performed using SureSelect Human All Exon V5 (Agilent Technologies, Santa Clara, CA). The exon‐enriched DNA libraries were subjected to paired‐end sequencing on a Hiseq2000 platform (Illumina, Inc., San Diego, CA). Sequence data were mapped with BWA and SAMTOOLS onto the hg38 human genome as a reference. Calls with variant quality less than 20 were filtered out and 95% of the targeted bases were covered sufficiently to pass our thresholds for calling single‐nucleotide polymorphisms (SNP) and small insertions or deletions (indels; Otogenetics Corporation, Norcross, GA). In order to confirm the variants, fragments containing the *MME* variants were amplified for direct Sanger sequencing in the patient II:2 and all family members available.

### Additional patients screening

To validate whether *MME* variants are associated with dHMN, genetic testing was performed on an additional 83 dHMN patients with unknown genetic cause using targeted next‐generation sequencing panel for inherited neuromuscular disorders that included the *MME* gene. These patients were recruited from 10 medical centers between January 2014 and December 2018. The clinical information was listed in Table S1.

### RNA transcript study

RNA was extracted using the RNeasy Mini Kit (Qiagen, Duesseldorf, Germany) according to the manufacturer's instructions from the blood lymphocytes of three patients, two carrier subjects (I:1 of family A and I:1 of family B), and a normal control participant. The extracted RNA was converted to cDNA using the SuperScript First‐Strand Synthesis System (Life Technologies, Carlsbad, CA) with random hexamer primers. The RNA concentration was measured using a NanoDrop1000 (Thermo Scientific, Waltham, MA). To evaluate the transcript result of the c.1416+2T>C variant, a fragment between the exon 11 and exon 15 of the cDNA of *MME* (ENST00000460393.5) was amplified by polymerase chain reaction (PCR) using primers of MME‐11F (5′‐TGGATCTTGTAAGCAGCCTCA‐3′) and MME‐15R (5′‐TCCACCTTTTCTCGGAGCTT‐3′). The PCR products were analyzed by agarose gel electrophoresis and Sanger sequencing.

The mammalian pCAG‐T7pol plasmids with CMV enhancer/chicken beta actin promoter (gift from Dr. Zhongming Liu) harboring wild‐type *MME* or mutant *MME* (c.1342C>T) were transfected into HEK293 cells for their overexpression. To explore the presence of nonsense‐mediated mRNA decay, cells were split into two subcultures. One subculture remained untreated, whereas the other one was exposed to 150 mg/mL cycloheximide (Sigma‐Aldrich, Santa Clara, CA) at 37°C for 4 h. After incubation, the cells were washed with Dulbecco's phosphate‐buffered saline (Thermo Scientific) and subsequently harvested by centrifugation.

Quantitative PCR (qPCR) was utilized for relative quantification of *MME* transcripts level. cDNA (10 ng) from each sample was amplified using a Fast SYBR Green master mix (Life Technologies) with primers for *MME* (F: 5′‐TGATCGCACTCTATGCAACC‐3′; R: 5′‐GCTCCCAGTTTTCTGTTGCT‐3′) and beta‐actin (F: 5′‐CCTCGCCTTTGCCGATCC‐3′; R: 5′‐GGATCTTCATGAGGTAGTCAGTC‐3′). The qPCR data were analyzed by the ViiATM7 PCR system (Life Technologies). Measurements were normalized against the beta‐actin gene. Levels of mutant transcript from the patients were quantified relative to the level of wild‐type transcript from a control. An unpaired t‐testing was used to compare the normalized and relative mean ΔCt values between the patients and control subjects. Statistical analyses were performed using SPSS software.

### MME expression and activity measurement

Sural nerve biopsy was performed on the patient II:2 of family A. Semithin sections of the specimen for light microscopy were stained with toluidine blue. The expression of MME protein in sural nerves was detected by immunohistochemical stain using mouse monoclonal MME antibody (UM870128, OriGene Technologies, Rockville, MD).

The sural nerve biopsy samples were homogenized, separated on a SDS‐PAGE gel, and then transferred to nitrocellulose membrane. The Nitrocellulose strips were probed with antibody against MME (UM870128, OriGene Technologies). The membrane was subsequently incubated with horseradish peroxidase‐conjugated secondary antibody (Chemicon, Temecula, CA). Chemiluminescent signals were digitally imaged (ECL‐Plus, Amersham, Little Chalfont, Buckinghamshire, UK).

The cDNA encoding human wild‐type *MME* was amplified by PCR and subcloned into pCAG‐T7pol plasmid through the Kpnl and Xbal endonuclease. The plasmids expressing the mutants c.1342C>T, c.1884C>G, c.2027C>T, and c.2071_2072delGCinsTT were generated by site‐directed mutagenesis according to the manufacturer's procedure (Takara MutantBEST Kit, Tokyo, Japan). HEK293 cells were cultured in DMEM with 10%v/v fetal bovine serum. Cells were transfected using Lipofectamine 2000 (Invitrogen, Carlsbad, CA) according to the manufacturer's procedure. After 24 h, cells were incubated with 100 μL reaction mixture containing 10 μmol/L of the MME substrate fluorescent Mca‐PLGL‐Dpa‐AR‐NH_2_ (R&D Systems, Minneapolis, MN). Fluorescence signal intensity of the cell supernatant was measured using a PekinElmer FL 8500 reader (Waltham, MA) with excitation at 320 nm and emission at 405 nm. Quantitative experiments were performed in triplicate and results were presented as mean ± standard deviation. For statistical analysis, one‐way ANOVA with Bonferroni post hoc test was used.

## Result

### Clinical features

The index patient was a 54‐year‐old woman from a nonconsanguineous family A (Fig. 1A II:2). She initially had muscle weakness of lower limbs at the age of 50. She complained of slow walking, muscle soreness, and falling down due to an uneven ground. Subsequently, she started having muscle weakness and tremors in both hands. Physical examination revealed mild atrophy of the intrinsic muscles of her feet and hands (Fig. 1C). Arthrogryposis or joint contracture was not detected in the lower limbs, but interphalangeal joint contracture of the left hand little finger was noted. Her muscle strength grade was 5/5 (Medical Research Council Scale) in the proximal limbs, 3/5 in the extensor digitorum brevis, 3/5 in the foot dorsiflexion, and 4/5 in the foot plantarflexion, respectively. Deep tendon reflexes were diminished in both lower limbs. Pain, light touch, vibration, and joint position sensations were normal. Babinski sign was negative on both sides. The clinical manifestations were summarized in Table 1.

**Table 1:** Clinical features of the patients with *MME*‐related dHMN.

Variable	Family A II:2	Family A II:5	Family B II:2
Sex	female	male	female
Age at onset (year)	50	45	16
Age at examination (year)	54	50	24
Symptoms at onset	Weakness of LL	Weakness of LL	Weakness of LL
Muscle wasting			
Upper limbs	Mild	mild	moderate
Lower limbs	moderate	moderate	severe
Muscle weakness^a^			
Upper limbs	+	+	++
Lower limbs	++	++	+++
Postural tremor	+	−	+
Finger joint contraction	+	−	+
Pes cavus	−	+	+
Babinski reflexes	−	−	−
Knee reflexes	+	−	−
Ankle reflexes	−	−	−
Sensory symptoms	−	−	−
MMSE score	30/30	29/30	30/30

MME, membrane metalloendopeptidase; dHMN, distal hereditary motor neuropathy; LL, lower limb; MMSE, Mini‐mental State Examination.

a Patients with distal muscle weakness, −: normal; +: <4 grade in distal muscles; ++: ≥2 grade in distal muscles; +++: <2 grade in distal muscles.

The younger brother of index patient was 50 years of age (Fig. 1A II:5). He first reported mild motor disability of the lower limbs at the age of 45, and hand weakness at the age of 49. Physical examination revealed distal motor weakness, wasting in the lower limbs and left hand, and pes cavus (Fig. 1D). Deep tendon reflexes were absent in the lower limbs. Pain, light touch, vibration, and joint position sensations were intact. There was no evidence of ataxia, tremor, or pyramidal tract signs. Other family individuals available were normal in the clinical evaluation.

The third patient was a 24‐year‐old woman from a nonconsanguineous family B (Fig. 1B II:2). She was identified in a group of 83 dHMN patients with unknown genetic cause. She had experienced a slowly progressive gait disturbance of the lower limbs since 16 years old. She presented with insidious wasting and weakness of distal lower limbs involving the foot dorsiflexors more severe than the plantar flexors, and developed foot drop and pes cavus (Fig. 1E). Progressive wasting of hand intrinsic muscles and disability to straighten her fingers became obvious at 22 years of age. Deep tendon reflexes were absent in the lower limbs. Pain, light touch, vibration, and joint position sensations were intact. There was no evidence of ataxia, tremor, or pyramidal tract signs.

### Electrophysiological study

The motor nerve conduction velocities were moderately reduced, but the amplitudes of compound muscle action potentials severely decreased. Furthermore, the motor nerves of lower limbs were more severely affected than those of upper limbs. The sensory nerve conduction velocities and action potentials were not affected in all three patients. Detailed nerve electrophysiological data are listed in the Table 2. For all three patients, needle electromyogram revealed distal denervation featured by the increased amplitude and duration of motor unit action potential during slight contraction, and reduced recruitment on voluntary contraction recorded in distal limbs.

**Table 2:** Electrophysiological studies in patients with *MME*‐related dHMN.

Motor nerve	Family A II:2			Family A II:5			Family B II:2		
	MNCV (m/sec)	dL (msec)	CMAP (mV)	MNCV (m/sec)	dL (msec)	CMAP (mV)	MNCV (m/sec)	dL (msec)	CMAP (mV)
L Median									
E‐W	50.0		10.1	46.0		4.2	48.6		4.9
W‐APB		3.3	9.8		3.5	5.1		3.4	6.7
R Median									
E‐W	52.6		7.5	50.0		7.2	50.0		8.2
W‐APB		3.8	6.7		3.7	8.9		3.8	8.9
L Ulnar									
E‐W	51.5		6.5	43.0		10.3	42.0		4.3
W‐ADM		2.4	7.0		2.0	12.1		2.4	4.1
R Ulnar									
E‐W	56.0		5.0	51.2		10.1	41.0		2.5
W‐ADM		2.7	4.3		2.7	11.2		2.6	3.2
L Peroneal									
FH‐A	40.8		1.1	39.0		0.5	37.5		0.3
A‐EBD		4.4	0.8		4.5	0.5		5.5	0.6
R Peroneal									
FH‐A	41.2		0.8	38.8		0.8	35.0		0.7
A‐EBD		4.7	1.0		4.8	0.7		4.8	0.9
L Tibial									
PF‐A	33.3		0.4	29.0		0.2	28.5		0.3
A‐AA		4.5	0.2		5.0	0.1		5.1	0.0
R Tibial									
PF‐A	29.4		0.1	38.0		0.2	30.3		0.2
A‐AA		4.7	0.0		4.6	0.3		4.6	0.1

Sensory nerve	SNCV (m/sec)	dL (msec)	SNAP (μV)	SNCV (m/sec)	dL (msec)	SNAP (μV)	SNCV (m/sec)	dL (msec)	SNAP (μV)
L‐Median									
IIIF‐W	60.8	2.3	76.7	56.7	2.6	72.8	58.8	2.5	67.5
R‐Median									
IIIF‐W	55.7	2.6	83.2	60.7	2.4	80.1	51.5	3.0	75.3
L Ulnar									
VF‐W	50.4	2.3	63.2	48.6	2.3	66.8	47.3	2.6	72.8
R Ulnar									
VF‐W	39.2	2.3	83.5	49.5	2.3	78.3	41.6	2.7	73.1
L‐Sural									
A‐SURA	50.0	2.9	8.5	51.0	2.8	8.8	43.7	3.5	8.1
R‐Sural									
A‐SURA	46.9	3.3	9.2	48.3	3.0	8.9	49.3	2.8	8.6

MME, membrane metalloendopeptidase; dHMN, distal hereditary motor neuropathy; MNCV, motor nerve conduction velocity; CMAP, compound motor action potential; dL, distal motor latency; SNCV, sensory nerve conduction velocity; SNAP, sensory nerve action potential; E, elbow; W, wrist; APB, abductor pollicis brevis; ADM, abductor digiti minimi; PF, popliteal fossa; A, ankle; FH, fibula head; EDB, extensor digitorum brevis; AA, abductor allucis; IIIF, third finger; VF, fifth finger. SURA, sural; Normal values are given in brackets.

### Genetic findings

In the exome‐sequencing process, after quality control and coverage criteria were met, we started with a total of 50,162 SNP and 2623 indels. Since hereditary motor neuropathy is a rare disorder but has a clear phenotype, there was a low likelihood that a causal mutation in the patient II:2 of family A was present in control populations. We therefore filtered for novel variants by comparing our exome data to several databases including dbSNP build 132, the 1000 Genomes Project, ExAC database, GnomAD, Hapmap, YH project, and the National Heart, Lung, and Blood Institute Exome Sequencing Project. After the exome capture sequencing and variant analysis, 318 variants under the dominant mode (220 SNP and 98 indels) and 52 variants under the recessive mode (41 SNP and 11 indels) were identified.

**Figure 2:**
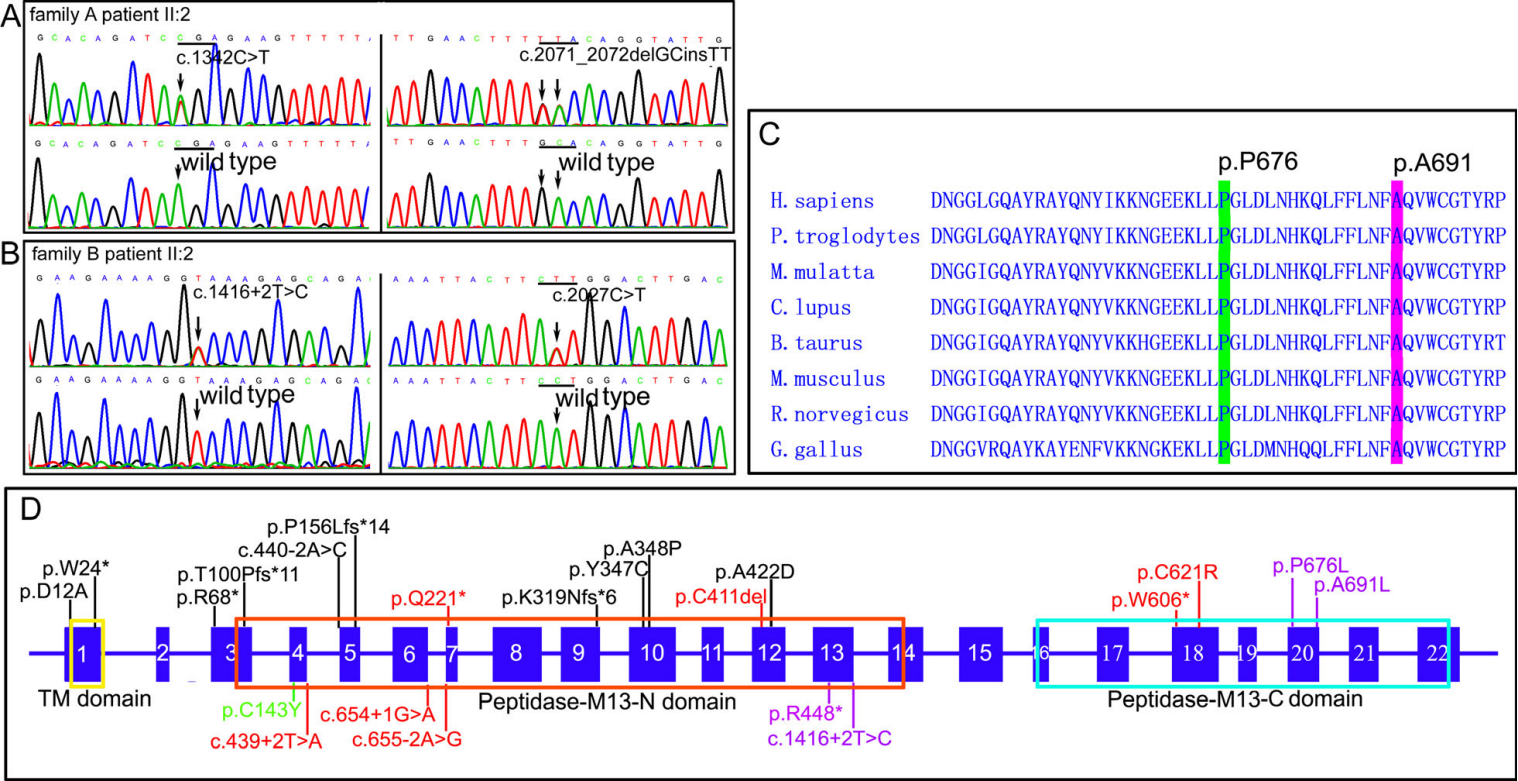
The genetic variants in the *MME* gene. (A) Electrophoregrams show that the patient II:2 of family A has compound heterozygous variants (variants at upper; wild‐type at below). (B) Electrophoregrams show that the patient II:2 of family B has compound heterozygous variants (variants at upper, wild‐type at below). (C) Residues proline 676 and alanine 691 have high evolutionary conservations. (D) Schematic representation of the *MME* gene and distribution of mutations reported. The mutations in *MME* are associated with dominant CMT2 (black font), recessive CMT2 (red font), dominant SCA43 (green font), and recessive dHMN (blue font). MME, membrane metalloendopeptidase; CMT2, Charcot‐Marie‐Tooth neuropathy; SCA43, spinocerebellar ataxia with neuropathy.

Comparing the candidate genes with the reported genes related to inherited peripheral neuropathies, we discovered that *MME* was on the candidate gene list with compound heterozygous variants of c.1342C>T (p.R448*) and c.2071_2072delGCinsTT (p.A691L). Sanger sequencing confirmed the heterozygous variants in the patient II:2 (Fig. 2A) and patient II:5 of family A. The variant of c.1342C>T was from the father, and the variant of c.2071_2072delGCinsTT was from the mother. The genetic screening in the family further demonstrated that the genotype co‐segregated with the phenotype (Fig. 1A). In an additional case series of 83 patients with dHMN, one patient (patient II:2 of family B) with compound heterozygous variants of c.1416+2T>C (p.V440_K472del) and c.2027C>T (p.P676L) in the *MME* gene were identified (Fig. 2B). Sanger sequence revealed that the variant of c.1416+2T>C was from the father, and the variant of c.2027C>T was from the mother, indicating a family co‐segregation (Fig. 1B).

The novel variants c.1416+2T>C, c.2027C>T, and c.2071_2072delGCinsTT have not been found in the 1000 genomes database, ExAC database, or gnomAD database, whereas the variant of c.1342C>T has been reported in patients with CMT.^7^ A homology search in different species demonstrated that the amino acids at the residues proline 676 and alanine 691 were highly evolutionarily conserved (Fig. 2C). The missense variants of p.P676L and p.A691L were predicted to be probably destructive with PolyPhen‐2 scores of 1.00, deleterious with a SIFT score of 0.00, and disease causing by MutationTaster, respectively. The donor site variant c.1416+2T>C changed the splice score from 0.91 to 0 predicted by NetGene2 server. The variants were considered as pathogenic according to the American College Medical Genetics guidelines.^15^ No other causative mutations associated with inherited peripheral neuropathies were found in the three patients.

### RNA expression analysis

**Figure 3:**
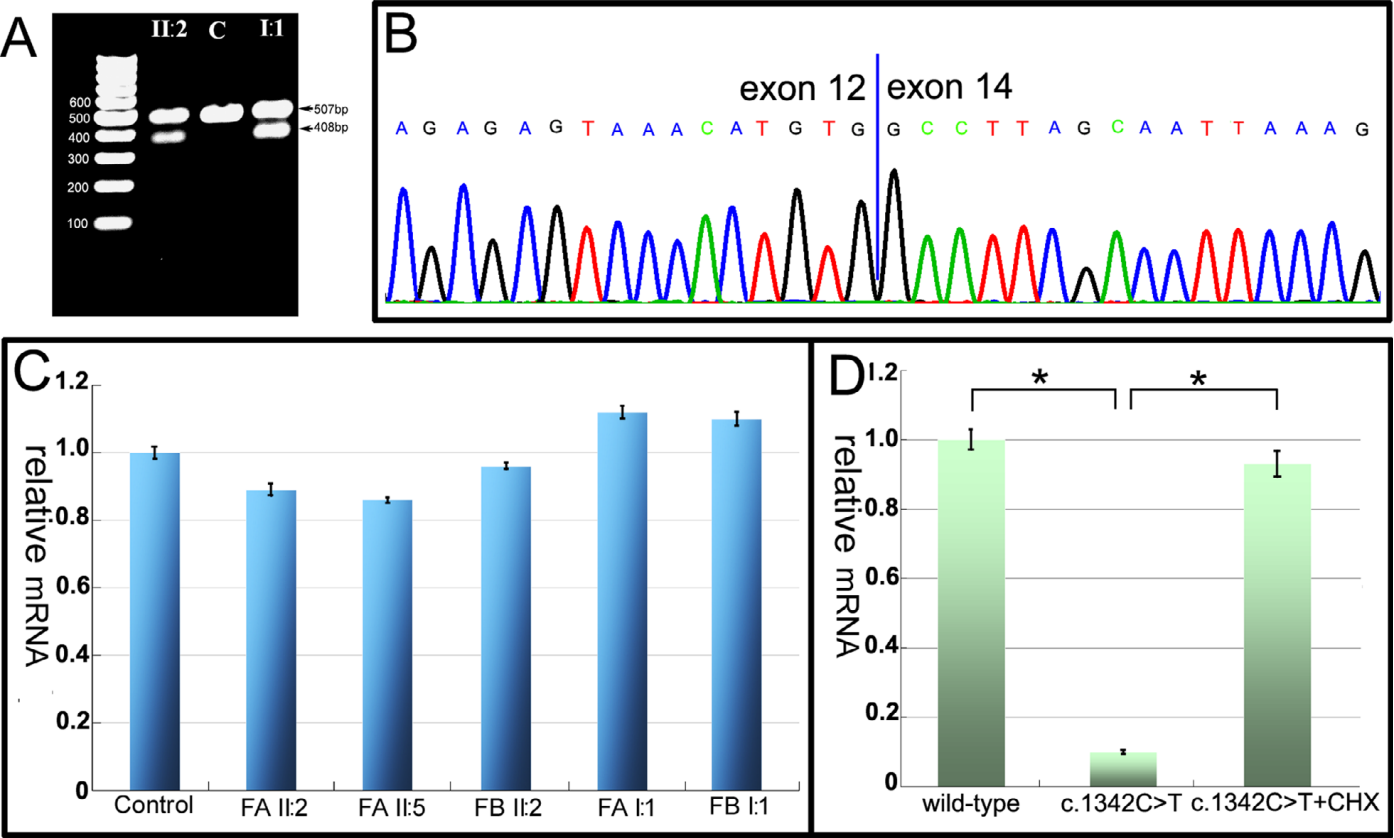
Genetic mutation analysis and RNA expression measurement. (A) Gel electrophoresis shows a loss 99‐bp fragment in the patient II:2 and her father (I:1) of the family B. (B) Electrophoregram of the RT‐PCR products reveals skipping of exon 13 in the MME transcript with c.1416+2T>C mutation. (C) Quantitative PCR showing mRNA levels of MME from patient and carrier lymphocytes relative to a control. Bars show the mean mRNA levels ± standard deviation relative to the control which has been set to +1. (D) HEK293 cells transfected with c.1342C>T mutant contain only about 10% *MME* mRNA compared to that of wild‐type cells, but the transcript of mutant *MME* is recovered after administration of cycloheximide (*P < 0.001). Bars show the mean mRNA levels ± standard deviation relative to the wild‐type which has been set to +1. MME, membrane metalloendopeptidase; PCR, polymerase chain reaction.

To evaluate the outcome of the novel donor site variant, RNA analysis was conducted on the patient II:2 of family B. The sequences of RT‐PCR products revealed aberrantly spliced mRNA lacking exon 13, resulting in a loss of 99‐bp fragment (Fig. 3A). The skip of exon 13 leads to an in‐frame deletion of 33 amino acids (p.V440_K472del) from the extracellular catalytic domain of MME (Fig. 3B). Premature stop variants might induce mRNA degradation via nonsense‐mediated mRNA decay. The mRNA level of MME in the lymphocytes mildly decreased in the three patients compared to a control, but no significant difference was identified between each other (Fig. 3C). However, the HEK293 cells transfected with c.1342C>T mutant contained only about 10% *MME* mRNA compared to that of wild‐type cells (Fig. 3D). Transcript levels could be increased after treatment with the translation inhibitor cycloheximide which blocks nonsense‐mediated mRNA decay.

### Histopathological findings and expression of neprilysin

**Figure 4:**
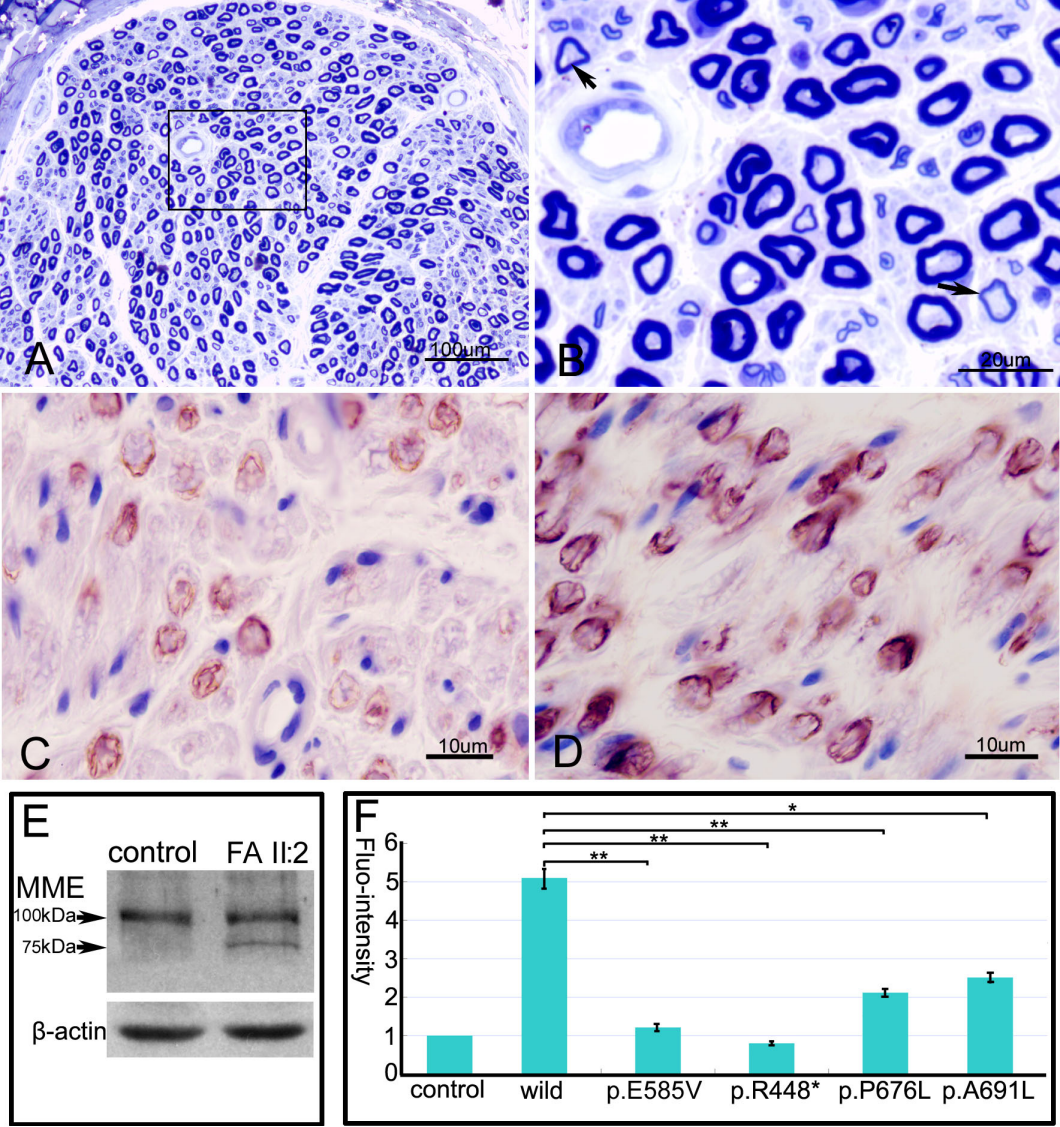
Histopathological findings and expression of MME. (A) Semithin section of sural nerve from the patient II:2 of family A shows a relatively normal density and structure of nerve fibers. (B) The magnification of square in the fig. A reveals few fibers with thin myelin sheaths (arrows). Mild decrease in positive reaction to the nerve fibers in the patient (C) compared to a control (D) by anti‐MME monoclonal antibody. (E) Immunoblot reveals a normal band and a truncated band in the homogenates of sural nerve with c.1342C>T premature mutant. (F) The disease‐associated MME mutants have significant decreases of MME enzymatic activity compared to the wild‐type (*P < 0.05, **P < 0.001). Bars show the mean levels ± standard deviation relative to the empty vector which has been set to +1. MME, membrane metalloendopeptidase.

A biopsy of the sural nerve was obtained from patient II2 of family A. Semithin sections showed relatively normal density and structure of large myelinated fibers, except very few fibers with thin myelin sheaths (Fig. 4A and 4). No axonal degeneration, clusters of myelinated fibers, or onion‐bulb formation were found. Immunohistochemical stain with an anti‐MME antibody showed a mild decrease in positive reaction to nerve fibers (Fig. 4C) compared to the sural nerve from the control (Fig. 4D). Immunoblot showed a normal 100kDa band and a truncated 75kDa band in the homogenates from the sural nerve (Fig. 4E), indicating a small amount of truncated protein still being produced, though a premature stop variant induced mRNA degradation through nonsense‐mediated mRNA decay.

### MME enzymatic activity

MME enzymatic activity was determined in HEK293 cells transiently transfected with plasmids encoding wild‐type human MME, disease mutants (c.1342C>T, c.2027C>T, and c.2071_2072delGCinsTT), and a proven mutant c.1754A>T (p.E585V) that results in the loss of NEP activity. As expected, the artificial control mutant almost abolished catalytic activity toward the substrate Mca‐PLGL‐Dpa‐AR‐NH_2_ compared with the background level of the empty vector control. The disease‐associated MME mutants showed significant decreases in MME enzymatic activity (Fig. 4F).

## Discussion

Distal HMNs are a group of clinically and genetically heterogeneous disorders caused by the dysfunctions of motor neurons in the peripheral nervous system (PNS). The phenotype of dHMN typically presents with a length‐dependent motor weakness and wasting, initially affecting the intrinsic foot muscles and the peroneal compartment of the leg, but most cases show variable involvement of the hand and proximal leg muscles as the disease progresses.^16^ In this study, the affected siblings presented with late‐onset muscle weakness and wasting of lower extremities, whereas the additional patient showed a juvenile‐onset phenotype of moderate muscle weakness and wasting involving both the upper and lower limbs. Although all patients presented with some extents of clinical heterogeneity, the clinical features were confined to pure axonal‐type motor neuropathy without sensory abnormalities. Therefore, these patients could be diagnosed as dHMN based on the clinical and electrophysiological evidence.

Family studies indicated that *MME* variants co‐segregated the phenotype in consistent with an autosomal‐recessive inheritance. Autosomal‐recessive dHMN is relatively rare and often clinically more severe.^17^ Patients with recessive dHMNs usually present with child‐onset distal extremity weakness and decrementation of life expency.^18^, ^19^ Our patients showed a late‐onset phenotype of distal motor symptoms, athough the juvenile‐onset patient had a moderate phenotype, which suggests that MME‐related dHMN may have a relatively benign clinical course.

The defects of MME have been identified in patients with autosomal dominant or recessive CMT2.^7^, ^9^, ^20^ Autosomal‐recessive variants of *MME* cause late‐onset CMT2, which displays as adult‐onset progressive weakness and atrophy of distal limb muscles, gait disturbance without wheelchair dependence, and sensory disturbance of the distal limbs.^10^ However, autosomal‐dominant variants of *MME* were linked to patients with late adult‐onset muscle weakness, muscle cramps, gait abnormalities with wheelchair dependence occasionally, and sensory disturbances.^7^, ^20^ Patients with heterozygous variants (c.466delC or c.674G>C) reported by Auer‐Grumbach *et al.* 7 or Lupo *et al.* 20 also showed muscle cramps in legs, fasciculation, distal lower limb weakness, stepagge gait, and very mild or negative distal sensory loss, suggesting that *MME* variants might be linked to a possibility of dHMN in those patients.^7^ dHMN shows apparent phenotypic overlap with CMT2, and also with proximal SMA, motor neuron diseases, hereditary spastic paraplegias, and other neurologic abnormalities.^12^ At least 10 genes (*AARS*, *BCSL2*, *DHTKD1*, *DYNC1H1*, *GARS*, *HSPB1*, *HSPB8*, *IGHMBP2*, *MFN2*, and *TRPV4*) have phenotypic overlaps between dHMN and CMT2.^2^ Therefore, it is possible that *MME* variants may also contribute to the etiology of conditions beyond CMT2, most plausibly dHMN. To the best of our knowledge, our study, for the first time, reported that recessive variants of the *MME* gene could be associated with dHMN. However, the identification of pathogenic mutations in the same gene associated with both dHMN and CMT2 indicates that these two diseases should belong to a continuum of MME‐related disorders.

MME is a zinc‐dependent metalloprotease that comprises a short N‐terminal cytoplasmic domain, a single transmembrane helix, and a large C‐terminal extracellular portion composed of two major alpha‐helical (peptidase M13) domains connected by a linker region (Fig. 2D).^21^ The variants associated with recessive CMT2 cause loss of MME expression.^10^ For example, the homozygous premature mutations of p.G179fs, p.G221*, p.C411del, c.439+2T>A, c.655‐2A>G, and compound heterozygous mutations of c.439+2T>A and c.655‐2A>G located in the N‐terminal peptidase M13 domain. The mutations of p.T606* and p.C621R located in the C‐terminal peptidase M13 domain would cause loss of function through nonsense‐mediated RNA decay or loss of a disulfide bridge with N‐terminal peptidase M13 domain, respectively. In our dHMN patients, the heterozygous variants p.R448* and p.V440_K472del are possibly associated with the loss of its enzyme activity of both N‐terminal and C‐terminal peptidase M13, but the variants p.P676L and p.A691L may only be associated with the function loss of enzyme activity of C‐terminal peptidase M13. Therefore, the loss of function in both N‐terminal and C‐terminal peptidase M13 might be related to motor CMT2 phenotype.^10^ However, the loss of C‐terminal peptidase M13 and partial preservation of N‐terminal peptidase M13 might be associated with dHMN phenotype.

MME has been found not only in the central nervous system (CNS), but also the PNS in mammals.^6^ MME has been well studied in the CNS for its β‐amyloid (Ab)‐degrading enzymatic activity, though elusive conclusions remains about its pathogenicity to Alzheimer's disease in humans.^22^ Although we are not aware of a direct link between MME and the maintenance of PNS, it is conceivable that MME, as a cleaving enzyme, might be responsible for the turn over molecules that are critical for the well‐being of peripheral nerves, or the degradation of peptides that have negative effects on neurons and peripheral nerves.^23^ The role of MME in PNS is unclear and warrants further investigation. The expression of MME in the sural nerve was significantly decreased in patients with autosomal‐recessive CMT2,^10^ while its expression was only mildly decreased in our patient, implying a subclinically biochemical alteration in sensory nerve, even though the patient currently presented with a dHMN phenotype. Therefore, a long‐term follow‐up is warranted in order to determine whether sensory disturbances would involve in the lower limbs of patients with autosomal‐recessive MME variants as the disease progresses.^24^

Our study indicates that variants in the *MME* gene are associated with autosomal‐recessive dHMN. The affected patients with MME‐related dHMN show a late‐onset phenotype with a relatively benign course. The expanding number of genes assigned to the “CMT2‐dHMN spectrum” should include the *MME* gene according to our observations.

## Author Contributions

D. H. contributed to the study design, data acquisition, analysis, and manuscript preparation. S. Y., P. F., J. C., X. Z., S. C., J. Z., D. T., L. W., X. H., Y. W., and M. L. contributed to data acquisition and analysis. L. X. and J. Z. gave critical suggestions on the manuscript writing. L. C. contributed to genetic analysis. S. Z. contributed to pathological analysis. H. O. contributed to experimental analysis. X. G. interpreted the data; revised the manuscript for intellectual content.

## Conflict of Interest

The authors report no disclosures relevant to the manuscript.

## Supporting information


**Table S1.** The clinical information of the additional 83 dHMN patients with unknown genetic cause.
