# Supplementary material for: Variants in MME are associated with autosomal‐recessive distal hereditary motor neuropathy
Source: Ann Clin Transl Neurol. 2019 Aug 20;6(9):1728–38. doi: 10.1002/acn3.50868 (PMC6764622; doi:10.1002/acn3.50868)
Supplement: Supplementary file 1 — Table S1. The clinical information of the additional 83 dHMN patients with unknown genetic cause. [file ACN3-6-1728-s001.doc]

**Table S1. The clinical information of the additional 83 dHMN patients with unknown genetic cause.**

| **patient** | **Age/sex** | **Main symptoms** | **Additional signs** | **PTR** | **pes cavus** |
| --- | --- | --- | --- | --- | --- |
| NC-1 | 54/F | dHMN | - | absent | - |
| NC-2 | 23/M | dHMN | - | reduced | + |
| NC-3 | 46/M | dHMN | pyramidal tract signs | brisk | - |
| NC-4 | 33/M | dHMN | - | absent | - |
| NC-5 | 69/F | dHMN | - | absent | - |
| NC-6 | 61/F | dHMN | vision loss | absent | - |
| NC-7 | 19/F | dHMN | - | absent | - |
| NC-8 | 17/F | dHMN | - | reduced | - |
| NC-9 | 40/M | dHMN | - | absent | - |
| NC-10 | 55/F | dHMN | - | absent | + |
| NC-11 | 39/F | dHMN | - | absent | - |
| HJ-1 | 71/F | dHMN | - | absent | - |
| HJ-2 | 68/M | dHMN | bulbar signs | absent | - |
| HJ-3 | 15/F | dHMN | - | reduced | - |
| HJ-4 | 36/F | dHMN | - | absent | - |
| HJ-5 | 41/F | dHMN | - | absent | - |
| HJ-6 | 47/M | dHMN | - | absent | - |
| HJ-7 | 39/F | dHMN | - | absent | - |
| HJ-8 | 60/M | dHMN | pyramidal tract signs | brisk | + |
| SZ-1 | 35/M | dHMN | - | absent | - |
| SZ-2 | 36/M | dHMN | - | reduced | - |
| SZ-3 | 61/M | dHMN | breathing difficulty | absent | - |
| SZ-4 | 20/M | dHMN | - | reduced | - |
| SZ-5 | 35/F | dHMN | - | absent | - |
| SZ-6 | 47/M | dHMN | - | absent | - |
| SZ-7 | 50/F | dHMN | - | absent | - |
| SZ-8 | 55/F | dHMN | bulbar signs | absent | + |
| SZ-9 | 34/M | dHMN | - | absent | - |
| SZ-10 | 68/M | dHMN | - | brisk | - |
| SZ-11 | 30/F | dHMN | - | reduced | - |
| SZ-12 | 25/M | dHMN | mild hearing loss | reduced | + |
| SX-1 | 60/F | dHMN | - | brisk | - |
| SX-2 | 34/F | dHMN | - | absent | - |
| SX-3 | 46/M | dHMN | - | absent | - |
| SX-4 | 46/M | dHMN | - | absent | - |
| SX-5 | 28/F | dHMN | mild numbness | absent | - |
| SX-6 | 17/F | dHMN | - | reduced | - |
| SX-7 | 26/M | dHMN | - | reduced | - |
| SX-8 | 36/F | dHMN | - | absent | + |
| SX-9 | 35/M | dHMN | - | reduced | - |
| SX-10 | 70/M | dHMN | - | absent | - |
| SX-11 | 54/F | dHMN | dysphagia and dysarthria | absent | - |
| SX-12 | 55/F | dHMN | - | absent | + |
| SX-13 | 63/F | dHMN | - | absent | - |
| SX-14 | 47/M | dHMN | bulbar signs | absent | - |
| SX-15 | 50/M | dHMN | - | absent | - |
| GY-1 | 13/F | dHMN | - | reduced | - |
| GY-2 | 29/F | dHMN | tremer | absent | - |
| GY-3 | 44/M | dHMN | - | absent | - |
| GY-4 | 50/M | dHMN | - | absent | - |
| GY-5 | 62/F | dHMN | dysarthria | absent | + |
| BT-1 | 49/F | dHMN | - | absent | - |
| BT-2 | 40/M | dHMN | - | absent | - |
| BT-3 | 43/M | dHMN | - | reduced | - |
| BT-4 | 30/F | dHMN | - | absent | - |
| BT-5 | 33/M | dHMN | - | reduced | + |
| BT-6 | 57/F | dHMN | - | absent | - |
| JJ-1 | 30/M | dHMN | - | absent | - |
| JJ-2 | 49/M | dHMN | - | absent | - |
| JJ-3 | 20/F | dHMN | mild hearing loss | reduced | - |
| JJ-4 | 26/M | dHMN | - | absent | - |
| JJ-5 | 39/F | dHMN | - | absent | - |
| JJ-6 | 65/M | dHMN | - | absent | - |
| JJ-7 | 58/F | dHMN | - | absent | + |
| LY-1 | 16/F | dHMN | - | reduced | - |
| LY-2 | 35/M | dHMN | - | absent | - |
| LY-3 | 66/F | dHMN | mild numbness | absent | - |
| LY-4 | 51/F | dHMN | ataxia | absent | - |
| KF-1 | 32/M | dHMN | - | brisk | - |
| KF-2 | 39/F | dHMN | - | absent | - |
| KF-3 | 64/F | dHMN | - | absent | + |
| KF-4 | 52/F | dHMN | - | absent | - |
| KF-5 | 28/F | dHMN | - | absent | - |
| BP-1 | 46/F | dHMN | pyramidal tract signs | brisk | - |
| BP-2 | 24/F | dHMN | - | absent | - |
| BP-3 | 65/M | dHMN | - | absent | - |
| BP-4 | 68/M | dHMN | - | absent | + |
| BP-5 | 57/M | dHMN | breathing difficulty | absent | - |
| BP-6 | 40/M | dHMN | - | absent | - |
| BP-7 | 48/F | dHMN | - | absent | + |
| BP-8 | 55/F | dHMN | dysarthria | absent | - |
| BP-9 | 24/M | dHMN | - | reduced | - |
| BP-10 | 38/M | dHMN | - | absent | - |
